# No association of xenotropic murine leukemia virus-related virus with prostate cancer or chronic fatigue syndrome in Japan

**DOI:** 10.1186/1742-4690-8-20

**Published:** 2011-03-17

**Authors:** Rika A Furuta, Takayuki Miyazawa, Takeki Sugiyama, Hirohiko Kuratsune, Yasuhiro Ikeda, Eiji Sato, Naoko Misawa, Yasuhito Nakatomi, Ryuta Sakuma, Kazuta Yasui, Kouzi Yamaguti, Fumiya Hirayama

**Affiliations:** 1 Department of Research, Japanese Red Cross Osaka Blood Center, 2-4-43 Morinomiya, Joto-ku, Osaka 536-8505, Japan; 2 Laboratory of Signal Transduction, Institute for Virus Research, Kyoto University, 53 Shogin Kawaharacho, Sakyo-ku, Kyoto 606-8507, Japan; 3 Department of Urology, Nishiwaki Hospital, 652 Shimotoda, Nishsiwaki, Hyogo 677-0043, Japan; 4 Department of Health Science, Kansai University of Welfare Science, 3-11-1 Asahigaoka, Kashiwara, Osaka 582-0026, Japan; 5 Department of Molecular Medicine, Mayo Clinic, College of Medicine, Rochester, MN55905, USA; 6 Laboratory of Viral Pathogenesis, Center for Human Retrovirus Research, Institute for Virus Research, Kyoto University, 53 Shogin Kawaharacho, Sakyo-ku, Kyoto 606-8507, Japan; 7 Department of Metabolism, Endocrinology and Molecular Medicine, Osaka City University Graduate School of Medicine, 1-4-3 Asahicho, Abeno-ku, Osaka 545-8585, Japan; 8 Department of Physiology, Osaka City University Graduate School of Medicine, 1-4-3 Asahicho, Abeno-ku, Osaka 545-8585, Japan; 9 Department of Molecular Virology, Tokyo Medical and Dental University, 1-5-45 Yushima, Bunkyo-ku, Tokyo 113-8519, Japan

## Abstract

**Background:**

The involvement of xenotropic murine leukemia virus-related virus (XMRV) in prostate cancer (PC) and chronic fatigue syndrome (CFS) is disputed as its reported prevalence ranges from 0% to 25% in PC cases and from 0% to more than 80% in CFS cases. To evaluate the risk of XMRV infection during blood transfusion in Japan, we screened three populations--healthy donors (*n *= 500), patients with PC (*n *= 67), and patients with CFS (*n *= 100)--for antibodies against XMRV proteins in freshly collected blood samples. We also examined blood samples of viral antibody-positive patients with PC and all (both antibody-positive and antibody-negative) patients with CFS for XMRV DNA.

**Results:**

Antibody screening by immunoblot analysis showed that a fraction of the cases (1.6-3.0%) possessed anti-Gag antibodies regardless of their gender or disease condition. Most of these antibodies were highly specific to XMRV Gag capsid protein, but none of the individuals in the three tested populations retained strong antibody responses to multiple XMRV proteins. In the viral antibody-positive PC patients, we occasionally detected XMRV genes in plasma and peripheral blood mononuclear cells but failed to isolate an infectious or full-length XMRV. Further, all CFS patients tested negative for XMRV DNA in peripheral blood mononuclear cells.

**Conclusion:**

Our data show no solid evidence of XMRV infection in any of the three populations tested, implying that there is no association between the onset of PC or CFS and XMRV infection in Japan. However, the lack of adequate human specimens as a positive control in Ab screening and the limited sample size do not allow us to draw a firm conclusion.

## Background

Xenotropic murine leukemia virus-related virus (XMRV), a gammaretrovirus found in humans, is possibly associated with certain diseases [1,2]. The virus was first identified in prostate cancer (PC) by using a pan-viral microarray; XMRV RNA was detected in eight of 22 R462Q homozygous patients, but in only one of 66 patients with RQ or RR (wild-type [WT]) alleles of the *RNASEL *gene [1], an important component of the innate antiviral response [3]. Schlaberg et al. [4] found XMRV proteins in nearly 25% of PC specimens and reported that XMRV infection is associated with high-grade PC. Conversely, XMRV RNA was detected in only 1.2% of PC cases in a German study [5], and neither XMRV RNA nor anti-XMRV antibodies (Abs) were detected in PC patients in another German cohort [6]. Furthermore, in a recent study, XMRV RNA was detected in the blood of 67% of patients with chronic fatigue syndrome (CFS) and 3.6% of healthy individuals [2]. Lo et al. [7] found murine leukemia virus (MLV)-related sequences in genomic DNA of peripheral blood mononuclear cells (PBMCs) in 32 of 37 (86.5%) CFS patients and three of 44 (6.8%) healthy blood donors. However, the absence of XMRV infection in CFS patients has been reported in several countries [8-12]. These conflicting results have provoked serious debates about XMRV detection methods and patient characteristics [13].

XMRV can infect many human cell lines by using XPR1 as a receptor, similar to other xenotropic murine retroviruses [14-16], and XMRV replication appears to be enhanced in cells with a defective interferon-gamma (IFNγ) intracellular pathway [17]. In terms of *in vivo *infection, the route of transmission, infectivity to humans, and pathogenesis of XMRV are largely unknown; therefore, its potential risk as a transfusion-transmissible infectious agent remains to be clarified.

Many blood service organizations worldwide, including those in Japan, have yet to establish a transfusion policy for XMRV, although in a few countries (e.g., Canada) blood donations are restricted from individuals previously diagnosed with CFS. To investigate the prevalence of XMRV in healthy Japanese individuals as well as in PC patients, we started screening blood samples in 2007 from donors in Osaka prefecture and PC patients in Nishiwaki City, a rural area of Hyogo prefecture close to Osaka prefecture, as a pilot study of XMRV infection. On the basis of Lombardi et al.'s results of XMRV infection in CSF patients and, to a lesser extent, in the healthy population [2], we also screened blood samples from CFS patients. We found that a proportion of the donors and patients had Abs against the XMRV Gag capsid (CA), but XMRV genes were barely detectable. These results suggest that although the presence of human infection with XMRV or XMRV-related viruses in Japan cannot be denied, such infection is likely to be limited.

## Results

### Study design

**Figure 1:**
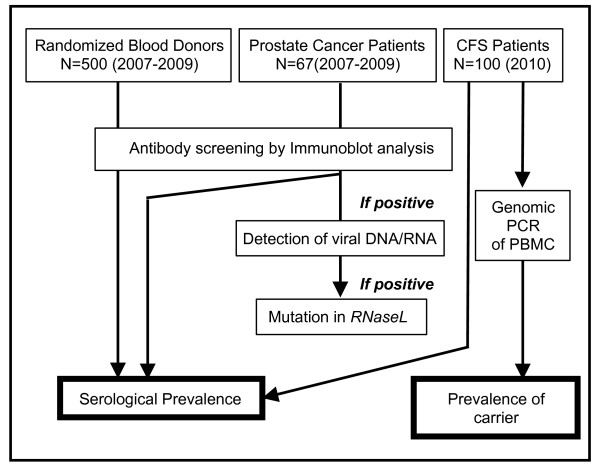
**Study flowchart**. Plasma samples randomly collected from 500 healthy donors, 67 PC patients and 100 CFS patients were screened for XMRV Abs in an immunoblot assay to estimate the serological prevalence of the virus. Viral Ab-positive PC patients were further tested for the presence of viral RNA in their plasma; genomic DNA from PBMCs of XMRV RNA-positive patients was also tested for viral DNA and *RNaseL *mutations. CSF patients were screened by genomic PCRs at three independent laboratories.

Our study design, summarized in Figure 1, was not standardized because the screening process for donors and PC patients was not implemented simultaneously with that for CFS patients. We employed different methods to detect XMRV nucleic acids at different stages of the study, but the same Ab-screening test was used consistently throughout. All plasma samples were screened for XMRV Abs by immunoblot assay to calculate the serological prevalence of XMRV. Plasma samples of viral Ab-positive PC patients were further screened for XMRV RNA. Moreover, PBMCs of PC patients whose plasma was positive for XMRV RNA were examined for the presence of XMRV genes and for *RNASEL *mutations in genomic DNA [1,18]. Plasma samples of CFS patients were simultaneously screened for XMRV Abs and genomic DNA according to published methods [1,2,6]. We did not examine XMRV DNA or RNA in the donor blood samples because, at present, the Japanese Red Cross Society does not have consensus for the genetic analysis of donor blood samples for research purposes, except for the analysis of blood types.

### Screening for XMRV Abs

**Figure 2:**
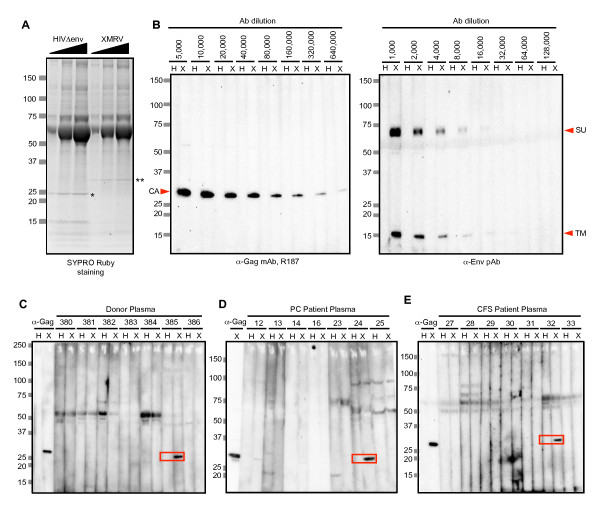
**XMRV Ab screening**. Immunoblot assay of proteins of HIV-1 Env-defective mutant (HIV ∆env) and XMRV clone VP62 for screening anti-XMRV antibodies in plasma. (A) Three different amounts of viral preparations (3, 6, or 9 μl/lane indicated by black triangles) were separated by 5-20% SDS-PAGE and stained with SYPRO Ruby. Asterisks represent Gag capsid (CA) proteins: *p24 in HIV and **p30 in XMRV. (B) Sensitivity of immunoblot assay used for screening. Viral lysates (3 μl) were detected with serially diluted control antibodies. An anti-spleen focus-forming virus (SFFV) Gag mAb (clone R187, left) and anti-XMRV Env pAb (right) was used for detection of Gag or Env proteins. Concentrations of detecting limit of each antibody were 6.3 ng/ml (1:640,000) in R187 mAb and 1.1 μg/ml (1:8,000) in anti-Env pAb. H, HIV∆env; X, XMRV; CA, Gag capsid; SU, Env surface subunit; TM, Env transmembrane subunit. (C-E) Ab screening by immunoblot assay of blood donor samples (C), PC patients (D), and CFS patients (E) using 3 μl of each viral lysate. Pairs of strips were incubated with 1:100 diluted plasma from individuals. XMRV-specific reactivity of substantial intensity was defined as a positive reaction (red squares).

To examine Abs against XMRV by immunoblotting, concentrated viral particles were used as antigens. When the same volume of XMRV and human immunodeficiency virus (HIV)-1 lysate as a negative control was analyzed by sodium dodecyl sulfate polyacrylamide gel electrophoresis (SDS-PAGE) and gel staining, we observed a comparable amount of Gag CA proteins in each preparation (Figure 2A, asterisks). The minimum amount of each virus lysate in which CA protein was detectable by gel staining with SYPRO ruby (3 μl) was used to assess sensitivity of the immunoblot assay by end point dilutions of an anti-Gag monoclonal antibody (mAb) (clone R187; Figure 2B, left) or an anti-Env rabbit polyclonal antibody (pAb) (Figure 2B, right). The detection limit of the screening assay was estimated as 6.3 ng/ml (1:640,000) for R187 mAb and 1.1 μg/ml (1:8,000) for anti-Env pAb.

In the Ab screening, we observed many nonspecific signals. Most of these reacted with both strips at the same mobility, and some weak bands were occasionally detected on either XMRV or HIV-1, or both strips at the position of the CA proteins, probably because of a large amount of CA protein on the strips. Therefore, we regarded such nonspecific signals as false positives, and considered that a band observed on the XMRV strip, but not on the HIV-1 strip, showing signal intensity comparable with that detected using the control anti-Gag mAb was positive for XMRV when the strips were blotted with 100 times-diluted plasma samples (red squares in Figure 2C-E). We identified 12 positive plasma samples: eight from the donors, two from PC patients and two from CFS patients. The prevalence of XMRV calculated from the immunoblot assay was 1.6% in blood donors, 3.0% in PC patients, and 2.0% in CFS patients (*p *> 0.05). Because XMRV was originally identified in PC samples [1], we analyzed whether there was a gender difference in the prevalence of XMRV; however, no significant difference between male and female subjects was noted (Table 1).

**Table 1:** Summary of anti-Gag Ab reactivities in study population

Population	Gender	Ab negative	Ab positive	Total	Prevalence (%)
Healthy donors	M	336	5	341	1.5
	F	156	3	159	1.9
	Total	492	8	500	1.6
Patients with PC	M	65	2	67	3.0
Patients with CFS	M	31	0	31	0
	F	67	2	69	2.9
	Total	98	2	100	2.0

No significant differences in prevalence were observed between the donors and the patients with PC and between the donors and the patients with CFS. Further, there were no significant differences in prevalence between the male and the female donors.

### Characterization of screening-positive Abs

**Figure 3:**
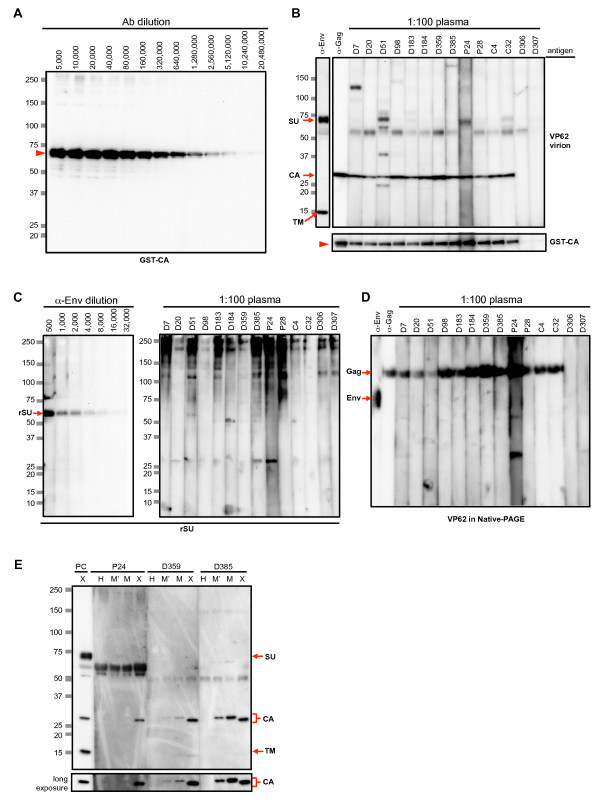
**Characterization of Gag CA-positive plasma samples**. (A) Sensitivity of immunoblot assay with GST-fused recombinant Gag CA (GST-CA) protein. GST-CA protein (300 ng per lane) was analyzed by 5-20% SDS-PAGE and detected with serially diluted R187 anti-Gag mAb. The concentration of the detection limit was 0.78 ng/ml (1:5,120,000). (B) Immunoblot assay of plasma samples that tested positive (D7, D20, D51, D98, D183, D184, D359, D385 in blood donors; P24 and P28 in PC patients; C4 and C32 in CFS patients) or negative (D306 and D307 in blood donors) for the screening immunoblot assay with 3 μl of VP62 virus lysate (upper panel) or 300 ng of the GST-CA recombinant protein (lower panel). For positive control, 8.5 μg/mL (1:1,000) of anti-Env pAb and 0.8 μg/ml (1:5,000) of anti-Gag mAb, R187, were used. (C) Immunoblot assay using recombinant Env SU (rSU) protein of xenotropic MLV. The detection limit of 300 ng of rSU protein was 1.1 μg/ml (1:8,000) by anti-Env pAb (left). One hundred diluted plasma samples tested positive for the screening assay were negative for rSU protein (right). (D) Immunoblot assay in a native-PAGE using 5 μl of the concentrated VP62 lysate in native sample buffer. Plasma samples testing positive (D7 to C32) and negative (D306 and 307) for the screening assay were examined. α-Env, anti-Env pAb (1:200, 42.5 μg/ml); α-Gag, R187 mAb (1:80,000, 50 ng/ml). (E) MoMLV particles with (M) or without (M') amphotropic Env were produced and subjected to an immunoblot assay to examine their cross-reactivity with XMRV-positive plasma. PC, a mixture of anti-Gag mAb (R187, 0.4 μg/ml) and anti-Env pAb (8.5 μg/ml) as the positive control. Arrow head, GST-fused Gag Capsid protein; SU, Env surface subunit; rSU, recombinant Env surface subunit of xenotropic MLV; TM, Env TM subunit; CA, Gag capsid protein.

Because we observed Abs against only the Gag CA protein in the Ab-screening assay, we examined test plasma for reactivity against recombinant Gag and Env proteins (Figure 3A-3C). For recombinant Gag protein, we expressed glutathione S transferase (GST)-fused Gag CA protein of XMRV derived from 22Rv1 cells. The sensitivity of the immunoblot assay using the GST-CA protein was about eight times higher than that used in the screening assay (Figure 3A, 1:5,120,000 dilution corresponding to 0.78 ng/ml R187 mAb). All screening-positive plasma, but not screening-negative plasma, tested positive for GST-CA proteins (Figure 3B), suggesting that the screening-positive plasma specifically recognized XMRV CA. In the upper panel of Figure 3B, D51, P24 and C32, plasma shows some signals migrating close to that of the Env surface subunit (SU). However, these were likely to be nonspecific as we observed similar signals on the paired HIV strip at the same position in the screening immunoblot assay (data not shown for D51, and Figure 2D and 2E for P24 and C32, respectively). We examined the reactivity of the test plasma against a recombinant histidine-tagged Env surface subunit protein (rSU) of a xenotropic MLV [19], in which the detection limit determined by endpoint dilutions was 1.1 μg/ml (1:8,000 dilution in Figure 3C, left), but detected no Abs against the Env SU protein in plasma samples (Figure 3C, right). An immunoblot assay after native-PAGE was also negative for Abs against Env proteins (Figure 3D). Detection limits in the native-PAGE were 6.3 ng/ml for anti-Gag mAb (R187) and 8.5 μg/ml for anti-Env pAb (data not shown).

To examine the specificity of the screening-positive plasma samples, we performed an additional immunoblot assay against proteins from Moloney murine leukemia virus (MoMLV), which has approximately 83% amino acid homology in the Gag region with XMRV. We observed multiple patterns of cross-reactivity (Figure 3E). Most screening-positive plasma samples were recognized exclusively with XMRV Gag CA (e.g., patient 24 in Figure 3E), but some showed weak cross-reactivity with Gag CA of MoMLV (donor 359 in Figure 3E). In another case, almost the same level of signal was detected against Gag CA of XMRV and MoMLV (donor 385 in Figure 3E). Plasma that predominantly reacted with MoMLV Gag was not observed. The Ab specificities are summarized in Table 2.

**Table 2:** Cross-reactivities with MoMLV proteins

Population	(-)	(+)
Healthy donors	5	3
Patients with PC*	1	
Patients with CFS	1	1
Total	7	4

The XMRV Ab-positive cases were categorized as having (+) or not having (-) cross-reactivities with Gag proteins of MoMLV.

*Cross-reactivity was not examined in one Ab-positive patient with PC (P28) because additional plasma from this patient was not available.

The serological prevalence of XMRV calculated using only the highly specific Ab was 1.0% in the donors, 1.5% in PC patients, and 1.0% in CFS patients. Again, there were no statistically significant differences in prevalence between blood donors and patients with either PC or CFS. We are unable to determine whether the anti-Gag CA Abs we identified would indicate XMRV infection or not, until panel plasma or serum samples collected from human subjects definitely infected with XMRV become available. Therefore, we tentatively regard those individuals who retain these Abs as suspicious cases.

### Detection of XMRV RNA in the plasma of PC patients

**Figure 4:**
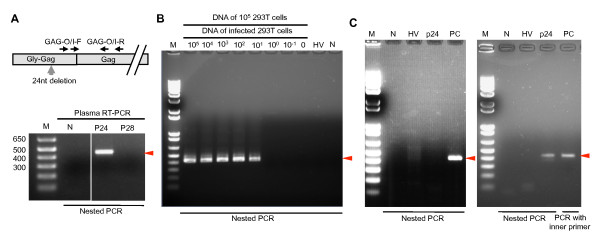
**Detection of XMRV genes from viral Ab-positive PC patients**. (A) Primer positions used in the PCR assay (upper panel). Gly-Gag; homologous region to glycosylated Gag of MLVs at the NH_2 _terminus of Gag. RNAs purified from the plasma of viral Ab-positive PC patients (P24 and P28) were used in a nested RT-PCR with primers GAG-O-F/R and GAG-I-F/R. Unnecessary lanes between the negative control without template RNA (N) and P24 have been removed from the original image (lower panel). (B) The detection limit of nested genomic PCR. Genomic DNA extracted from serially diluted 293T cells infected with 22Rv1 cell-derived XMRV (indicated as 10^5^~0) was mixed with genomic DNA extracted from 10^5 ^293T cells. For one reaction of PCR with a volume of 20 μl, 100 ng of each DNA mixture was used. The final concentration of viral genome contained in a PCR reaction was calculated as 7610.5-0.152 cell equivalents of genomic DNA from 293T cells infected with 22Rv1 cell-derived XMRV (corresponding to the lanes indicated as 10^5^-10^-1 ^of infected 293T cells). The detection limit of the nested PCR was calculated as approximately 1.5 cell equivalents (indicated as "10^1^"). (C) Inconsistent results of nested genomic PCR tests for XMRV using genomic DNA extracted from PBMCs. In a 20 μL volume, 100 ng genomic DNA were used for amplification. Nested genomic PCRs were performed on September 17 (left) and September 18 (right), 2008. M, molecular size marker; N, negative control without nucleic acids; P24 and P28, nucleic acids purified from PBMCs of P24 or P28; HV, genomic DNA of healthy volunteer; PC, diluted XMRV VP62 plasmid; arrow head, amplified band using inner primer pair.

In April 2008, we examined XMRV RNA from the plasma of two screening-positive PC patients (P24 and P28) by nested RT-PCR: only one patient (P24) had positive results for XMRV RNA with Gag-specific primers (Figure 4A). The sequence of the amplified PCR product was 99.8% (412/413), identical to that of XMRV VP62 (data not shown). However, we could not conclude that the PCR product was derived from XMRV infection because this fragment did not contain an XMRV-specific 24 nucleotide deletion in the *gag *region [1]. The patient's malignant prostate tissue was not available because it had already been removed and was not deposited in the hospital.

**Figure 5:**
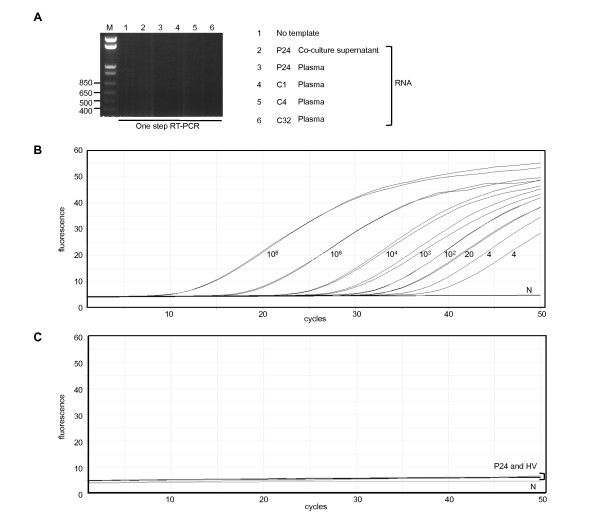
**Detection of XMRV RNA and DNA in viral Ab-positive samples**. (A) RNA was purified from 1 mL of coculture supernatant of activated PBMCs and LNCap-FGC cells (lane 2) or 1 ml plasma (lanes 3-6). For one-step RT-PCR, 15 μl of 60 μl eluted RNA was amplified in a 25 μl volume. CFS patients C4 and C32 tested positive for XMRV Abs but C1 was negative. (B) Detection of XMRV *env *by TaqMan real-time PCR assay. Duplicated test samples of diluted XMRV plasmid (VP62) were amplified. The detection limit of the TaqMan real-time PCR was 4 copies/reaction determined by VP62 plasmid. (C) Duplicated test samples without template DNA in negative control (N) or with genomic DNA extracted from PBMCs of a viral Ab-positive PC patient (P24) and healthy volunteers (HV) were amplified as for (B).

In August 2008, we collected whole blood from this patient to examine *RNASEL *mutations at amino acid positions 462 [1,18] and 541 [20], and found a WT residue at 462 and a low-risk amino acid residue (Glu) at 541 (data not shown). We tried to isolate infectious or full-length XMRV from PBMCs of this patient, but were unsuccessful. We also found that the test results of the nested PCR assay, in which detection limit was approximately 1.5 cell equivalents of genomic DNA from 293T cells infected with 22Rv1 cell-derived XMRV (Figure 4B), using PBMC-extracted genomic DNA were not reproducible (Figure 4C). In November 2009, the whole blood of P24 became available again and was tested for XMRV DNA and RNA. Although the plasma still tested positive for Abs against XMRV Gag CA, neither XMRV RNA nor DNA was detected with the same method used in April 2008 (data not shown). We further examined XMRV RNA from plasma and supernatants of co-cultured P24 PBMCs with LNCap-FGC cells using one-step RT-PCR, but both tested negative for the XMRV Gag gene (Figure 5A). We performed real time PCR on genomic DNA extracted from PBMCs, which is capable of amplifying a fragment of the Env gene with a detection limit of four copies/reaction, but the additional PCR tests of P24 were negative for the XMRV gene (Figure 5B and 5C). These data suggested that the amount of XMRV in the blood of the Ab-positive PC patient was limited, if the virus still existed. Alternatively, it remains possible that the results of the original P24 PCR tests were false positive.

### Detection of XMRV DNA in PBMCs of CFS patients

**Figure 6:**
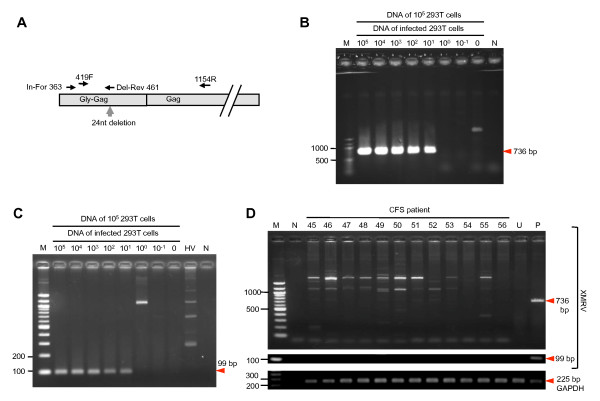
**Screening of CFS patients using genomic PCR**. (A) Primer positions used in the PCR assay. Gly-Gag; homologous region to glycosylated Gag of MLVs at the NH_2 _terminus of Gag. The detection limits of the genomic PCR assays with primers 419F and 1154R, and In-For 363 and Del-Rev 461 are shown in (B) and (C), respectively. Genomic DNA extracted from serially diluted 293T cells infected with 22Rv1 cell-derived XMRV was mixed with genomic DNA extracted from 1.0 × 10^5 ^293T cells. For one reaction with a volume of 20 μl, 100 ng of each DNA mixture was used. The final concentration of the detected viral genome was calculated as 7610.5-0.152 copies (corresponding to the lanes of 10^5^-10^-1 ^infected 293T cells, respectively) in a reaction. The detection limit of both PCR tests is approximately 1.5 cell equivalents of genomic DNA from 293T cells infected with 22Rv1 cell-derived XMRV indicated as "10^1^"of infected 293T cells. (D) Representative results of PCR assay with primers indicated in (B) (upper) and (C) (middle). The human GAPDH gene was examined as an internal control (bottom). M, molecular size marker; HV, genomic DNA of healthy volunteers; N, no template; U, genomic DNA of uninfected 293T cells; P, genomic DNA of infected 293T cells.

To examine the prevalence of XMRV in CFS cases, we screened CFS patients for XMRV DNA in PBMCs at three independent laboratories. Figure 6 shows the representative results with two primer sets. The sensitivities of our PCR tests with primer sets indicated in Figure 6A were determined using genomic DNA extracted from 293T cells infected with 22Rv1 cell-derived XMRV (Figure 6B and 6C). The detection limit of both PCR tests was calculated as approximately 1.5 cell equivalents of genomic DNA from 293T cells infected with 22Rv1 cell-derived XMRV. In screening PCR tests, we observed several nonspecific bands but the XMRV gene was not amplified as shown in Figure 6D. Although bands of a similar size to that expected were occasionally observed, sequencing analysis indicated that they contained human genomic DNA rather than XMRV genes (data not shown).

In the Japanese Red Cross Osaka Blood Center, we performed nested RT-PCR analysis of the *gag *region by using plasma RNA (Figure 5A), and a real-time TaqMan PCR assay of genomic DNA to amplify the *env *region (data not shown) if the patients tested positive for Abs. We observed no positive results from the PCR assays performed at the three independent laboratories or this additional PCR test, indicating that there were no detectable amounts of XMRV DNA in the blood of CFS patients, although two of 100 patients tested positive for the XMRV Gag Ab (Figure 2E, 3B, and 3D, and Table 1).

## Discussion

In this study, we identified a small number of people who possessed Abs against XMRV Gag CA, regardless of gender or disease condition (PC and CFS), but none of the individuals in the three tested populations retained strong Ab responses to multiple XMRV proteins. We were unable to isolate XMRV from the blood of PC patients and detected no XMRV genes in the blood of any CFS patients.

We screened blood donors and patients with PC and CFS for XMRV Abs using a similar method to that developed as our in-house confirmatory test for human T-lymphotropic virus (HTLV)-1 infection in Japanese blood donors in the late 1980s, as no XMRV-positive human plasma was available to validate XMRV Ab tests. Unlike HTLV and HIV infection, XMRV-positive plasma bound only to Gag CA proteins in our study. However, in feline gammaretrovirus infections, immune responses are not always strong enough to induce a detectable amount of Abs [21]. In an animal study of XMRV infection, Qiu and colleagues [22] found that rhesus macaques intravenously inoculated with 3.6 ×10^6 ^50% tissue culture infective dose of XMRV showed good Ab responses against Env SU, Env transmembrane subunit (TM), and Gag proteins. In this animal model, transient viremia was observed for less than 2 weeks, but the Ab responses prolonged over 100 days post-inoculation and declined thereafter without boosting, despite high-dose viral inoculation [22]. These data suggest that XMRV replication is relatively limited *in vivo *to induce lasting immune responses compared with HIV and HTLV infection. Alternatively, the anti-Gag CA Abs we observed could account for cross-reactivity with other immunogens, although seven of 11 Ab-positive plasma samples showed high specificity to XMRV Gag (Figure 3E and Table 2). In addition, Western blotting of 2262 blood donors by Qiu and colleagues identified two blood donors positive for anti-p30 (CA) Ab and one positive for anti-gp70 (Env SU) [22]. These Ab-positive blood donors showed no multiple reactivities to viral antigens, as observed in the present study, but the prevalence of the single antigen-reactive donor was much lower than that in our current result (0.13% vs. 1.6%, respectively). It is possible that the positive reaction to CA protein might include more cross-reactivity in our study. Further investigation of human plasma collected from individuals clearly infected with XMRV is required to verify our Ab screening results.

At the beginning of our study, the presence of XMRV in the blood of PC patients had not been reported; however, we speculated that XMRV might infect blood cells similar to the infection of PBMCs by other gammaretroviruses [23]. We obtained positive nested RT-PCR results on plasma collected from the Ab-positive PC patient only with extensive PCR conditions of 50 cycles using outer and inner primer pairs (Figure 4A, P24). We were, however, unable to consistently detect the XMRV gene in the same patient 4 and 15 months later using freshly collected blood samples. Co-cultivation of activated PBMCs by Concanavalin A and IL-2 with the LNCap-FGC cell line, which is highly susceptible to XMRV [17], gave rise to devastating LNCap-FGC cell death (data not shown), and we were unable to detect XMRV genes in the cell culture (Figure 5A). Our data suggest that P24 was perhaps infected with XMRV or some related viruses, but viral replication in the blood was somewhat limited. If this is the case, the prevalence of XMRV in PC patients (one of 67 patients) would be relatively close to that previously reported [5]. We cannot, however, exclude the possibility that the positive P24 signal in the PCR assays was caused by contamination, as discussed recently [24-26]. We did not PCR-amplify mouse-derived genetic materials [24,25] because of the lack of remaining P24 test sample that tested positive for XMRV PCR, although we did use a hot start Taq polymerase that is inactivated not by anti-Taq mouse mAbs but by chemical modification in our RT-PCR test [26].

We were unable to detect XMRV DNA or RNA in CFS patients, in accordance with the results of some previous studies [8-12]. It is unlikely that our detection procedures caused such a big difference from those studies that reported a prevalence of 67% or 86.5% [2,7], because all studies employed highly sensitive PCR methods. The difference may instead be explained by the characteristics of patient populations. All CFS patients in our study met the Centers of Disease Control and Prevention (CDC) diagnostic criteria [27]; however, the currently employed diagnosis of CFS is not based on objective and quantitative measures but on the claims of patients and some authorized criteria.

Although our results of Ab screening are ambiguous, we conclude that XMRV infection is not involved in the onset and/or progression of PC and CFS in the population we screened. Even if the Abs we detected, or at least the XMRV-specific ones, were caused by XMRV infection, there was no statistically significant difference in the serological prevalence of XMRV among the three populations of the study. Moreover, the negative or inconsistent PCR results in the Ab-positive patients can be explained by the limited replication of XMRV *in vivo*. Alternatively, by assuming that the Ab reaction is attributable to cross-reactivity, the negative PCR results likely indicate the absence of XMRV infection in patients. In either case, our results do not support an association between XMRV and CFS, in line with previous findings [8-12].

Retroviral integration is theoretically harmful to the host cell because it disrupts the host genome. To reduce the risk of XMRV infection during blood transfusion, a reliable screening strategy should be established. The implementation of such a screening or inactivation protocol for blood products, however, will be influenced by the evaluation of the prevalence of XMRV by a universal test with high sensitivity and specificity, which must be urgently developed.

## Conclusions

Our data for Japanese blood donors, PC patients and CFS patients imply that there is no association between the onset of PC or CFS and XMRV infection, although the lack of adequate human specimens as a positive control and the limited sample size do not allow us to draw an ultimate conclusion.

## Methods

### Sample collection

Plasma samples randomly collected from healthy donors (*n *= 500) at the Japanese Red Cross Osaka Blood Center between December 2006 and May 2009 were subjected to XMRV Ab screening. All donors had negative results in the routine tests at the Center: antigen testing of hepatitis B virus (HBV) and human parvovirus B19; Ab testing against HBV, hepatitis C virus (HCV), HIV-1, HIV-2, HTLV-1, and syphilis; nucleic acids of HIV-1, HIV-2, HBV, and HCV. All procedures in the donor screening study were performed according to the guidelines of the Japanese Red Cross Society, which do not permit the detection of nucleic acids from unapproved viruses.

All patients with PC enrolled in this study (*n *= 67) received medical treatment at Nishiwaki City Hospital (Hyogo Prefecture, Japan) between December 2007 and December 2009, when plasma samples were collected, and provided written informed consent. Whole blood samples in ethylenediaminetetraacetic acid (EDTA) were separated by centrifugation, and the plasma was stored at -80°C until use. PBMCs of the patients who tested positive for XMRV Abs and RNA were used for *RNASEL *sequencing and viral isolation. This study was approved by the ethical committee of Nishiwaki City Hospital.

CFS patients in this study fulfilled the 1994 CDC Fukuda criteria [27] and received medical treatment at the Fatigue Clinic Center, Osaka City University Graduate School of Medicine, Osaka, Japan between April and August 2010. Most of the patients were female (69%) with an age distribution of 17-62 years (mean, 38 years). The mean interval from disease onset to blood collection was 126.5 months (11-337 months). Duplicated tubes of 4 ml of whole blood in EDTA were used for Ab screening and genomic PCR assay. Whole blood samples were also collected into sodium heparin tubes (Becton Dickinson, Franklin Lakes, NJ) for cell culture. All blood samples were conveyed to the Japanese Red Cross Osaka Blood Center and genomic DNA was purified from them on the same day. Three aliquots of genomic DNA purified from one patient were independently analyzed at three laboratories. This study was approved by the Ethics Committee of Osaka City University Graduate School of Medicine and all blood samples were collected with written informed consent.

### Cell lines and culture

Human 293T and 22Rv1 cells were obtained from the American Type Culture Collection (CRL-1537 and CRL-2525, respectively; ATCC, Manassas, VA). Human prostate cancer cell line LNCap-FGC was obtained from the RIKEN Cell Bank (Tukuba, Japan), and the GP293 packaging cell line was purchased from Clontech Laboratories (Mountain View, CA). These cells were grown in Dulbecco's modified essential medium supplemented with 10% fetal bovine serum (FBS) and antibiotics. Rat hybridoma cell line R187 was obtained from ATCC (CRL-1912) and maintained in RPMI-1640 medium supplemented with 50 nM 2-mercaptoethanol, 10% FBS, and antibiotics. Before collecting the culture supernatant, the growth medium was replaced with CD Hybridoma medium (Invitrogen, Carlsbad, CA) supplemented with 8 mM l-glutamine. For recombinant Env production, Sf9 and High Five cells (Invitrogen) were maintained in Sf-900 III SFM and Expressed Five medium (Invitrogen), respectively.

### Control antibodies

IgG proteins in culture supernatants from R187 cells, prepared against SFFV Gag and able to react with Gag capsid proteins from a wide variety of gammaretroviruses [28], were purified using a protein G affinity column (MabTrap Kit; Amersham Biosciences, Piscataway, NJ). For anti-Env Abs, rabbits were immunized with a mixture of two peptides (PRVPIGPNPV[C] of Env SU and [C]QFEQLAAIHTDLG of Env TM; [C] indicates an additional cysteine residue for peptide purification), and their antisera were collected and purified after five immunization steps with a Protein A affinity column (GE Healthcare, Buckinghamshire, UK). Concentrations of the purified R187 mAb and anti-Env pAb were 4.0 mg/ml and 8.5 mg/ml, respectively.

### Antibody screening

An infectious XMRV molecular clone, pcDNA3.1-VP62, was provided by Dr. R. H. Silverman. To produce the viral particles, 293T cells were transfected with pcDNA3.1-VP62 by a liposome method (Lipofectamine LTX; Invitrogen). Two days after transfection, the culture supernatant was collected, filtered, and concentrated 20 times by centrifugation at 20,000 × *g *for 4 h at 4°C. The concentrated virus was suspended in a Laemmli SDS sample buffer. As a negative control, we prepared an env-defective HIV-1 virus (pNL∆env, provided by Dr. A. Adachi) by using the same method as for XMRV. A MoMLV-derived retrovirus vector was produced using the GP293 cell line, with or without transfection of an amphotropic Env expression vector (provided by Dr. D. R. Littman). Viral proteins were separated by 5-20% gradient SDS-PAGE and either stained with SYPRO Ruby (Bio-Rad, Hercules, CA) or transferred to a polyvinylidene difluoride membrane (Wako Pure Chemical Industries, Osaka, Japan) cut into strips. After blocking with 5% skimmed milk in Tris-buffered saline (TBS), the strips were incubated with 1:100 diluted donor or patient plasma samples at 4°C overnight. After washing with TBS containing 0.05% Tween-20, the strips were incubated with 1:5,000 diluted horseradish peroxidase (HRP)-conjugated anti-human IgG Ab (GE Healthcare), and detected by ECL Plus (GE Healthcare). For endpoint dilutions, a pair of strips was blotted with 0.8 μg/ml-6.25 ng/ml (1:5,000-1: 640,000) R187 mAb and detected using 1:5,000 diluted HRP-conjugated anti-rat IgG (H+L) secondary Ab (Jackson ImmunoResearch Laboratories, West Grove, PA) for Gag, or blotted with 8.5 μg/ml-66.4 ng/ml (1:1,000-1:128,000) anti-Env pAb and detected using 1:2,500 diluted HRP-conjugated anti rabbit IgG (GE Healthcare).

### Other immunoblot assays

To produce GST-fused XMRV Gag CA protein, a 789-bp fragment of the CA gene was amplified using genomic DNA of 293T cells infected with XMRV derived from 22Rv1 cells, and cloned into the pET-42b(+) vector (Merck KGaA, Darmstadt, Germany). The GST-CA protein was purified by a Glutathione-Sepharose 4B column (GE Healthcare) from bacterial lysate of BL21 Star (DE3) (Invitrogen) transformed by the GST-fused CA expression plasmid. To produce His-tagged recombinant Env SU of xenotropic MLV [19], a PCR-amplified *env *SU region was cloned into pcDNA3.1myc/His (Invitrogen) followed by subcloning of an env-His DNA fragment into the Bac-to-Bac Baculovirus Expression System (Invitrogen). The supernatant of Sf9 cells transfected with the bacmid was used for infection of HighFive cells. Recombinant Env proteins collected from the culture supernatant of infected cells were purified using a HisTrapHP column (GE Healthcare). In the native-PAGE, concentrated viruses were suspended with native sample buffer (Native Sample Buffer; Bio-Rad) and separated on a 5-20% gel in a Tris-glycine buffer (25 mM Tris-Cl, 192 mM glycine, pH 8.4). The subsequent procedures were for the Ab-screening immunoblot assay.

### Detection of viral nucleic acids

For RT-PCR analysis of Ab-positive PC patient samples (Figure 4A), RNA was isolated from 500 μl of plasma using the PureLink Viral RNA/DNA Kit (Invitrogen), and 8 μl of the 10 μl eluted RNA was reverse-transcribed using Superscript III (Invitrogen) with random hexamer primers in a total reaction volume of 10 μl. In the nested PCR assay, 3 μl cDNA or 100 ng genomic DNA of PBMCs was amplified in a 20 μl volume with primer pairs GAG-O-F/R and GAG-I-F/R [1] and AmplyTaq Gold DNA polymerase (Applied Biosystems, Foster City, CA) for 50 cycles. The PCR cycling conditions were as follows: activation at 95°C for 5 min; then 50 cycles of 95°C for 15 s, 60°C for 15 s, and 72°C for 60 s (30 s in the second-round PCR); with a final extension at 72°C for 7 min.

To extract genomic DNA from CFS patients, 4 ml of whole blood in EDTA were centrifuged at 1500 × *g *for 10 min at room temperature, and 200 μl of the buffy coat were transferred to a 2 ml tube for DNA purification using the QIAamp Blood Mini Kit (Qiagen GmbH, Hilden, Germany). We divided 180 μl of eluted DNA equally into three tubes for analysis at three independent laboratories: Department of Research, Japanese Red Cross Osaka Blood Center, and the Laboratories of Signal Transduction and Viral Pathogenesis, Institute for Virus Research, Kyoto University, Japan. PCR of 1 μg genomic DNA in a 50 μl reaction was performed with primer pairs GAG-O-F/R and GAG-I-F/R [1] for nested genomic PCR (data not shown) or 419F and 1154R [2] and In-For363 and n-Rev536 [6] for single PCR. In the genomic PCRs, we used PrimeSTAR GXL DNA polymerase (Takara Bio, Shiga, Japan) with the following conditions: activation at 98°C for 2 min; then 45 cycles of 98°C for 10 s, 63°C for 15 s, and 68°C for 45 s; and a final step at 68°C for 2 min. For one-step RT-PCR (Figure 5A), RNAs were purified from 1 ml of 4-day culture supernatants of P24 PBMCs activated with 10 ng/ml concanavaline A (J-Oil Mills, Tokyo, Japan) and 100 U/ml IL-2 (e-Bioscience, San Diego, CA) and maintained with LNCap-FGC cells or patient plasma using a QIAamp Ultrasense Virus Kit (Qiagen). One-step RT-PCR was performed using 15 μl of 60 μl eluted RNA and a 419F and 1154R primer pair [2] and the following conditions: reverse transcription at 50°C for 30 min; activation at 95°C for 15 min; then 45 cycles of 94°C for 30 s, 57°C for 30 s, and 72°C for 1 min; and a final extension at 72°C for 10 min.

TaqMan real-time PCR tests were performed with 200 ng of genomic DNA, Universal ProbeLibrary, and FastStart TaqMan Probe Master (Roche, Basel, Switzerland) in a total reaction volume of 20 μl with a Rotor-Gene Q thermal cycler (Qiagen). Primer and probe sequences are as follows: 5'-cctagtggccaccaaacaat-3' (Env forward), 5'-ggccccaaggtctgtatgta-3' (Env reverse), and 5'-FAM-gctccagg-3' (Env probe, #1 of Universal ProbeLibrary). The following condition was used: 1 cycle of 95°C for 10 min, and 50 cycles of 95°C for 15 s and 60°C for 45 s.

### *RNASEL *mutation

In patients whose serum tested positive for XMRV RNA, mutations of *RNASEL *at amino acid positions 462 [18] and 541 [20] were examined as previously described [1,20]. PCR-amplified genomic DNA fragments were sequenced using an ABI PRISM 3100 genetic analyzer (Applied Biosystems).

### Statistics

Non-parametric analysis was performed with the Mann-Whitney *U*-test to determine any statistical significance in the data. A *p *value of less than 0.05 was considered to be significant.

## Abbreviations

Ab: antibody; ATCC: American Type Culture Collection; CDC: Centers of Disease Control and Prevention; CFS: chronic fatigue syndrome; EDTA: ethylenediaminetetraacetic acid; FBS: fetal bovine serum; HBV: hepatitis B virus; HCV: hepatitis C virus; HIV: human immunodeficiency virus; HRP: horseradish peroxidase; HTLV: human T-lymphotropic virus; IFNγ: interferon-gamma; MLV: murine leukemia virus; PAGE: polyacrylamide gel electrophoresis; PBMC: peripheral blood mononuclear cell; PC: prostate cancer; SDS: sodium dodecyl sulfate; TBS: Tris-buffered saline; XMRV: xenotropic murine leukemia virus-related virus; WT: wild-type.

## Competing interests

The authors declare that they have no competing interests.

## Authors' contributions

RAF conceived and designed the study, coordinated the collaboration, carried out the Ab screening and PCR tests, and drafted the manuscript. TM designed the study, coordinated the collaboration for studies of XRMV infection in CFS patients and attempted to isolate XMRV. TS recruited PC patients and carried out immunohistochemical testing of prostate tissues (data not shown). HK helped in designing the study and recruiting CFS patients. YI developed the real-time PCR test. ES conducted the Ab screening and PCR tests of CFS patients and attempted to isolate XMRV. NM conducted the PCR tests of CFS patients. YN and KY helped in designing the study and recruiting CFS patients. RS participated in the development of the real-time PCR test. KY participated in the Ab screening. FH helped in designing the study and drafting the manuscript. All authors have read and approved the final manuscript.
